# Thy-1-Integrin Interactions in *cis* and *Trans* Mediate Distinctive Signaling

**DOI:** 10.3389/fcell.2022.928510

**Published:** 2022-06-06

**Authors:** Ping Hu, Lisette Leyton, James S. Hagood, Thomas H. Barker

**Affiliations:** ^1^ Department of Biomedical Engineering, School of Engineering and Applied Science, University of Virginia, Charlottesville, VA, United States; ^2^ Cellular Communication Laboratory, Program of Cellular and Molecular Biology, Center for Studies on Exercise, Metabolism and Cancer (CEMC), Faculty of Medicine, Universidad de Chile, Santiago, Chile; ^3^ Advanced Center for Chronic Diseases (ACCDiS), Faculty of Chemical and Pharmaceutical Sciences and Faculty of Medicine, Universidad de Chile and Institute of Biomedical Sciences, Faculty of Medicine, University of Chile, Santiago, Chile; ^4^ Department of Pediatrics, Division of Pulmonology, School of Medicine, University of North Carolina, Chapel Hill, NC, United States; ^5^ Program for Rare and Interstitial Lung Disease, School of Medicine, University of North Carolina, Chapel Hill, NC, United States

**Keywords:** integrin, Thy-1 (CD90), *trans*-interaction, *cis*-interaction, signaling, pathophysiology

## Abstract

Thy-1 is a cell surface glycosylphosphatidylinositol (GPI)-anchored glycoprotein that bears a broad mosaic of biological roles across various cell types. Thy-1 displays strong physiological and pathological implications in development, cancer, immunity, and tissue fibrosis. Quite uniquely, Thy-1 is capable of mediating integrin-related signaling through direct *trans-* and *cis-*interaction with integrins. Both interaction types have shown distinctive roles, even when interacting with the same type of integrin, where binding in *trans* or in *cis* often yields divergent signaling events. In this review, we will revisit recent progress and discoveries of Thy-1–integrin interactions in *trans* and in *cis*, highlight their pathophysiological consequences and explore other potential binding partners of Thy-1 within the integrin regulation/signaling paradigm.

## 1 Introduction

Thy-1 [also known as cluster of differentiation 90 (CD90)] is a 25 kDa cell-surface glycoprotein located in lipid rafts, which is tethered to the outer leaflet of the cell membrane *via* a glycosylphosphatidylinositol (GPI) anchor. This protein was first identified more than half a century ago on mouse T cells (Reif and Allen 1964a). Thy-1 has been identified as a member of immunoglobin superfamily (IgSF), displaying strong homology to both the variable and constant regions of immunoglobin (Williams and Gagnon 1982). The protein is widely expressed among different cell types and across many species, and is involved in neurogenesis, immunity, development, fibrosis, and cancer. These functions have been previously reviewed elsewhere (Haeryfar and Hoskin 2004; Barker and Hagood 2009; Herrera-Molina, Valdivia et al., 2013; Leyton and Hagood 2014; Hagood 2019; Saalbach and Anderegg 2019; Sauzay, Voutetakis et al., 2019). In this review, we will focus on the *trans-* and *cis-*interactions between Thy-1 and other cell surface molecules, especially the interactions with the first identified Thy-1 receptors: the integrins.

The binding of Thy-1 to αvβ3 integrin was first reported as a *trans*-interaction of astrocytes with EL4 cells. The interaction is dependent on the RLD tripeptide, a structural analog of the established integrin binding RGD motif (Ruoslahti 1996) present on Thy-1, which triggers canonical integrin outside-in signaling (Leyton, Schneider et al., 2001). Subsequently, Thy-1 has been shown to interact with αvβ3 integrin in *cis*; this interaction is critically important for fibroblasts to appropriately sense and respond to the mechanical stiffness of their surroundings, *via* an essential functionality of this integrin known as mechanotransduction (Fiore, Strane et al., 2015). Collectively, existing evidence suggests Thy-1 as a dual-functional integrin regulator —mediating integrin downstream signaling through direct *trans*-interaction, while also regulating baseline integrin activity/avidity in *cis*-by preferentially coupling to integrin in its inactive conformation.

More specifically, *trans*-interaction between Thy-1 and integrin, which also involves the heparan sulfate proteoglycan syndecan-4 forms a tri-molecular complex that regulates the adhesion and migration of melanoma cells, blood cells and astrocytes. Importantly, a bidirectional communication has been described between astrocytes associated with other brain cells, such as neurons, where not only astrocytes migrate in response to Thy-1, but neurons also respond to integrin/syndecan-4 binding by contracting their elongated processes and acquiring a rounded shape (Burgos-Bravo, Martinez-Meza et al., 2020). On the other hand, the *cis*-interaction of Thy-1 with integrins also mediates a different biological role versus its *trans* interactions. The integrin binding RLD motif on Thy-1 can directly bind to integrin molecules within the same lipid raft and thus, constrain activation-independent extension of integrins, further stabilizing the bent-inactive conformation. In addition, through recruiting various lipid raft-bound proteins to the proximity of the integrin cytoplasmic tail, Thy-1 enables a cell to “feel” its environmental stiffness and react accordingly (Fiore, Strane et al., 2015). The details of the molecular basis and biological significance for both *trans* and *cis* interactions between Thy-1 and integrins will be carefully reviewed and discussed in the following sections.

## 2 Thy-1 Directly Binds Integrins in *Trans*

Thy-1–integrin interaction in *trans* was originally described *in vitro* in 2001, as the association of astrocytes containing αvβ3 integrin with a Thy-1^+/+^-thymoma cell line (EL-4), but not with Thy-1^−/−^- EL-4^−f^ cells, (Leyton, Schneider et al., 2001). Since then, evidence has indicated that more physiologically relevant cell-cell interactions mediated by this Thy-1–integrin interaction exist, including 1) activated endothelial cells and cancer cells; 2) activated endothelial cells and neutrophils, monocytes; 3) activated fibroblasts and dendritic cells; 4) fibroblasts and cancer cells; as well as 5) neurons and astrocytes. In all these cases, the *trans* cell-cell interaction could potentially trigger downstream signaling; however, detailed molecular mechanisms have not been deciphered for all the cells involved. In this section, we will review the cells that reportedly participate in these Thy-1–integrin interactions, the molecular mechanisms triggered downstream of these encounters and then, incorporate a third element known to contribute to this association: syndecan-4. Because αvβ3 integrin and syndecan-4 are both considered mechanoreceptors, we will also address the signaling mechanisms that are involved downstream of these receptors, when cells are subjected to external forces due to their binding to other cells through Thy-1.

### 2.1 Thy-1–Integrin Interaction in Cell-Cell Interactions

#### 2.1.1 Activated Endothelial Cells and Cancer Cells

The αvβ3 integrin–Thy-1 interaction mediates the binding of various cancer cells to endothelial cells (ECs). Reportedly, ECs do not express Thy-1, but they do so under inflammatory conditions. Thy-1 expression levels on the cell surface are enhanced *in vitro* by cytokines, such as Tumor Necrosis Factor (TNF), and Thy-1 is also detected in ECs of primary melanoma, but not in tissue sections obtained from benign skin lesions (Schubert, Gutknecht et al., 2013). On the other hand, melanoma cells are more aggressive with higher levels of activated αvβ3 integrin, since this integrin stimulates tumor growth and extracellular matrix (ECM) invasion (Johnson 1999). This is possible through the interaction of endothelial Thy-1 with the αvβ3 integrin in melanoma cells, since antibodies targeting these molecules inhibit melanoma *trans*-endothelial migration and the adhesion of melanoma cells to the human dermal microvascular endothelial cells (HDMEC) under flow or static conditions (Saalbach, Wetzel et al., 2005). In addition, using mouse B16F10 melanoma cells in an isogenic model of metastasis *in vivo*, Leyton and co-workers recently demonstrated that unlike melanoma cells expressing αvβ3 integrin, those lacking this integrin fail to metastasize to the lung (Brenet, Martinez et al., 2020). Importantly, another study that used Thy-1 knockout mice, showed that B16F10 cells (αvβ3 integrin^+/+^), injected *via* the tail vein do not metastasize (Schubert, Gutknecht et al., 2013). Moreover, reported that human breast cancer cells MDA-MB-231, as well as B16F10 melanoma cells, adhere to activated EC *in vitro*, allowing *trans*-endothelial migration of these cancer cells in a β3 integrin-dependent manner Brenet, Martinez et al. (2020). These *in vitro* results, together with those using cells with silenced β3 integrin in a metastatic model, highlight the importance of Thy-1–integrin interaction in cancer-endothelial cell interactions in melanoma and breast cancer progression and metastasis.

Thy-1 has been associated with tumor progression in several types of cancers. In malignant pleural mesothelioma, Thy-1 is found overexpressed in primary cancer cells obtained from tumors exposed to chemotherapeutic drugs *in vitro*. The elevated expression of Thy-1 correlates with tumor progression, which has been additionally associated with lower survival rate of patients according to data from The Cancer Genome Atlas (TCGA) (Oehl, Kresoja-Rakic et al., 2018). Thy-1 is also considered a tumor promoter in pancreas adenocarcinoma, where its expression is elevated in the stroma fibroblasts and in ECs, favoring tumor growth and angiogenesis (Zhu, Thakolwiboon et al., 2014). Additionally, Thy-1 and α6 integrin overexpression have been associated with high metastasis and poor survival in gallbladder cancer patients (Zhang, Yang et al., 2016). The α6 integrin, which pairs with two distinct β subunits to form the laminin binding integrins α6β1 and α6β4, has also been associated with metastasis in other cancer types, such as hepatocarcinoma and breast cancer, respectively (Carloni, Mazzocca et al., 2001; Yoon, Shin et al., 2006).

#### 2.1.2 Activated Endothelial Cells and Leukocytes

Thy-1 in HDMEC binds to αMβ2 integrin in leukocytes. Proinflammatory cytokines, such as TNF, can activate the ECs, inducing an increase in Thy-1 expression levels (Brenet, Martinez et al., 2020). Leukocytes could then associate with ECs through an αMβ2 integrin–Thy-1 interaction and undertake *trans-*endothelial migration, suggesting that leukocytes could reach distant organs through inflamed tissues by crossing barriers with activated ECs (Wetzel, Chavakis et al., 2004). Thy-1 in activated ECs could also bind to αMβ2 integrin in polymorphonuclear cells derived from patients with psoriasis and promote their transmigration through the EC layer to accumulate in the skin (Wetzel, Wetzig et al., 2006).

The αXβ2 is a different leukocyte integrin that has also been described as a Thy-1 receptor. Although a direct interaction of αXβ2 integrin with Thy-1 was validated more than 15 years ago through SPR experiments (Choi, Leyton et al., 2005), the biological function of this interaction has not yet been established. However, a recent study showed an interaction of a tumor promoter molecule, extracellular matrix protein1 (ECM1) with αXβ2 integrin, which induces cancer cell stemness through the phosphorylation of the AKT/FAK/paxillin/Rac pathway. The binding of ECM1 to αXβ2 integrin affects the ability of this integrin to bind to Thy-1, thus altering Thy-1 function. Additionally, overexpression of ECM1 or its silencing correlates with Thy-1 expression levels (Yin, Wang et al., 2021). Here, Thy-1 could account, in part, for the cancer cell stemness induced by the ECM1-αXβ2 integrin association because, on the one hand, there will be greater Thy-1 expression and, on the other, the Thy-1 integrin partner will be sequestered by the ECM1 binding; however, this is a possibility that remains to be investigated.

#### 2.1.3 Activated Fibroblasts and Dendritic Cells

Dendritic cells (DCs) enzymatically clear their way through the ECM, and with the aid of dermal fibroblasts reach the lymph. DCs and fibroblasts contact each other *via* the interaction of Thy-1 in fibroblasts and β2 integrin in DCs, in *vitro* cultures (Saalbach, Klein et al., 2007; Saalbach, Klein et al., 2010). Dermal fibroblasts increase the capabilities of DCs to migrate upon TNF/IL-1β treatment of fibroblasts. Boosted DC migration is attributed to the induction of membrane metalloprotease-9 (MMP-9) expression by the dermal DCs. Similarly, evidence indicates that lung fibroblasts can direct DC migration under inflammatory diseases, like chronic obstructive pulmonary disease and chronic asthma. These events are coordinated by αvβ8 integrin, which activates Transforming Growth factor-β (TGF-β) (Kitamura, Cambier et al., 2011); however, the participation of Thy-1 is unclear in this scenario. In a different study, αvβ5 integrin was described to lead to TGF-β activation through the processing of the latency-associated peptide (LAP), and it was shown that Thy-1 could regulate this process by binding to the integrin *in cis*, preventing its interaction with LAP (Zhou, Hagood et al., 2004). Therefore, it is tempting to speculate that Thy-1 could also play a role in the αvβ8 integrin-mediated activation of TGF-β in airway remodeling. Thus far, this possibility has not been studied.

#### 2.1.4 Fibroblasts and Cancer Cells

Interestingly, cancer-associated fibroblasts (CAFs) are known to promote tumor progression; however, the molecular mechanism by which CAFs regulate these events are unknown. One study has shown that gastric cancer patients with a large number of CAFs exhibit drug resistance and a poor prognosis. These CAFs produce extracellular vesicles that, when injected in a peritoneal metastasis mouse model, induce drug resistance. Additionally, these CAF vesicles, when added to cancer cells grown in Matrigel, lead to β1 integrin stabilization on the plasma membrane and drug resistance *in vitro* (Uchihara, Miyake et al., 2020). Another study indicated that CAFs align the fibronectin matrix through increased cellular contractility, generating traction forces that allow cancer cells to migrate directionally and invade tissues to promote metastasis (Erdogan, Ao et al., 2017). A different study in human lung adenocarcinoma indicated that Thy-1 is expressed in these CAFs and the presence of an elevated number of Thy-1^+/+^ CAFs is a sign of poor prognosis (Schliekelman, Creighton et al., 2017). Therefore, an interesting possibility is that, apart from aligning the ECM protein fibronectin, these Thy-1^+/+^ CAFs could guide integrin-positive cancer cells to migrate and metastasize. Indeed, several integrins have been involved in cancer cell migration over ECM proteins secreted by CAFs (for a recent review on this topic, see Jang and Beningo 2019); but in this case, the contribution of the Thy-1–integrin interaction to cancer cell migration and invasion has yet to be identified.

In ovarian cancer cells, the scenario is more complex since Thy-1 can exert both tumor promoter and suppressor functions. On the one hand, Thy-1^+/+^ cancer stem cells in ovarian cancer show a high proliferative and self-renewal capability compared to Thy-1^−/−^ cells. Additionally, Thy-1 knockdown in these cells makes the cancer features disappear (Leyton, Diaz et al., 2019). However, different studies have reported that Thy-1 is downregulated in ovarian cancer tissues and that Thy-1 overexpression decreases tumor formation *in vivo* and anchorage-independent growth *in vitro*, effects that are dependent on the expression of αvβ3 integrin (Abeysinghe, Cao et al., 2003; Chen, Hsu et al., 2016). The *in vitro* effects of Thy-1 overexpression are mirrored by silencing β3 integrin, indicating a role of Thy-1 in *cis*-regulation of this integrin. Therefore, as it is clear from other reported examples, the context-dependent role of Thy-1 (Bradley, Ramirez et al., 2009) could perhaps explain these differences in tumor regulation.

#### 2.1.5 Neurons and Astrocytes

The interaction of β3 integrin and Thy-1 was first described as an important trigger of morphological changes in astrocytes (Leyton, Schneider et al., 2001). Later, the interaction was shown to be involved in neuron-astrocyte association and to be mediated by the αvβ3 integrin (Hermosilla, Munoz et al., 2008). The binding of these two molecules was then held responsible for changes occurring in neurons, suggesting a bidirectional signaling emanating from each component: the αvβ3 integrin in astrocytes and Thy-1 in neurons. Importantly, this *trans*-interaction was challenged by the presence of αvβ3 integrin in neurons, posing the question as to whether the *cis-*interaction of Thy-1–integrin in neurons would play a role in the signaling triggered in *trans* by a similar integrin. By silencing neuronal β3 integrin, showed that the Thy-1–integrin interaction *in cis* is dispensable for αvβ3 integrin transactivation of neurite outgrowth inhibition and suggested that the neuronal αvβ3 integrin could bind Thy-1 and form small nanoclusters that would regulate the binding of the integrin with other cellular or ECM ligands (Maldonado, Calderon et al., 2017). Therefore, many interactions could occur in parallel between ECM-integrins and integrins and proteins present on the same (*cis*) or on different (*trans*) cells, supporting the idea of a complex regulation of the function of many cell adhesion molecules. Therefore, αvβ3 integrin binding to Thy-1 constitutes an important interaction in neurite-astrocyte communication. However, some studies have indicated that neither anti-integrin antibodies, RGD peptides, nor Thy-1-Fc protein can completely abolish the functionality of the αvβ3 integrin–Thy-1 interaction, suggesting that other molecules participate in the cellular outcome. Indeed, syndecan-4 was reported as a mediator of a trimolecular interaction with Thy-1 and αvβ3 integrin, and the complex is required for the formation of focal adhesions and stress fibers induced by the engaged receptors in astrocytes (Avalos, Valdivia et al., 2009). As suspected, the trimolecular complex formed was also important for the effect of Thy-1–integrin interaction in neurite retraction (Burgos-Bravo, Martinez-Meza et al., 2020). In this case, syndecan-4 accelerated the effect of Thy-1–integrin ligation, inducing faster cytoskeleton contraction, neurite retraction and inhibition of neurite outgrowth (Burgos-Bravo, Martinez-Meza et al., 2020).

### 2.2 Thy-1 Regulates Downstream Integrin Signaling Through *Trans*-interactions

As mentioned above, detailed molecular mechanisms downstream of Thy-1–integrin receptors are still ill defined; therefore, in this section we will summarize what has been best described thus far. Breast cancer and melanoma cells treated with Thy-1 trigger signals by engaging β3 integrin. The molecular cascade stimulated downstream of integrin ligation involves increased intracellular Ca^2+^ concentration, Connexin-43 and Pannexin-1 hemichannel opening, ATP release, and P2X7 receptor (P2X7R) activation. This signaling mechanism occurs in these two models: cancer cell migration and invasion. Considering that the transmigration of breast cancer and melanoma cells is significantly decreased when cells have low levels of β3 integrin and that, in a preclinical mouse model, melanoma cells that normally metastasize to the lung cannot reach the target organ when β3 integrin has been silenced (Brenet, Martinez et al., 2020), the signaling mechanism triggered by the Thy-1–integrin interaction is important for cancer cell migration, invasion and transvasation both in *in vitro* and *in vivo* models. The aforementioned signaling pathway triggered by Thy-1–integrin ligation was previously reported for activated astrocytes (Henriquez, Herrera-Molina et al., 2011; Alvarez, Lagos-Cabre et al., 2016; Lagos-Cabre, Alvarez et al., 2017).

Anti-αv integrin monoclonal antibodies, like CNTO 95 (also known as intetumumab), have demonstrated the important role of this integrin in tumor promotion and metastasis. CNTO 95 proved useful in inhibiting invasion of breast cancer cells (Chen, Manning et al., 2008), as a standalone treatment (Mullamitha, Ton et al., 2007) or in combination with other drugs in patients with prostate cancer (O'Day, Pavlick et al., 2011; O'Day, Pavlick et al., 2012). However, subsequent phase II studies of CNTO 95 showed no clinical benefits (Heidenreich, Rawal et al., 2013), resulting in the discontinuation of the trials [reviewed in (Rocha, Learmonth et al., 2018)]. It is conceivable that this failed anti-integrin strategy is at least partially caused by the presence of Thy-1, which is particularly highly-expressed in those invasive cancer cells (Sauzay, Voutetakis et al., 2019).

Dermal fibroblasts are activated by TNF/IL-1β treatment and produce IL-6 which then stimulates DCs to produce MMP9, helping these cells to migrate through the ECM and transmigrate through basement membrane-like structures (Saalbach, Klein et al., 2010). Dermal DCs co-cultured with activated fibroblasts produce 10-fold higher levels of MMP9 than DCs treated directly with the proinflammatory cytokines TNF/IL-1β. In addition, experiments using antibodies indicated that the high production of MMP9, but not MMP2, account in part for the enhanced motility of DCs in an inflamed tissue. Interestingly, the interaction of activated fibroblasts with neutrophils also promotes secretion of MMP9 by neutrophils, and in this case, the Thy-1–αMβ2 integrin interaction is required for MMP9 production (Saalbach, Arnhold et al., 2008). In the context of fibroblast-DC interaction, the Thy-1–integrin interaction might also play an important role in DC migration, since integrins are recognized players of cell migration. In this skin inflammatory model, IL-6 was recognized for the first time as a cytokine capable of inducing DC production of MMP9 and thus, as an important regulator of DC migration [see recent review on this topic in (Perez, Leyton et al., 2022)].

In gastric cancer cells growing in Matrigel, their exposure to extracellular vesicles produced by CAFs, leads to a rapid change of shape in these cancer cells. A proteomic analysis performed with these vesicles revealed the presence of Annexin A6 in CAF-vesicles, but not in cancer cell-vesicles. In this study, Annexin A6 is shown to stabilize β1 integrin, thereby leading to drug resistance. CAF-vesicles can activate focal adhesion kinase (FAK) and promote nuclear translocation of the yes-associated protein (YAP) in cancer cells. Therefore, ECM-ligated β1 integrin induces drug resistance through the activation of the FAK and YAP signaling pathways (Uchihara, Miyake et al., 2020). Importantly, extracellular vesicles reportedly play a role in the development of drug resistance by various mechanisms, which include transport of drugs, drug pumps, pro-survival cargos, and mRNAs (Burgos-Ravanal, Campos et al., 2021). It is noteworthy that Thy-1 has been shown to co-exist with Annexin A6 on extracellular vesicles (Morciano, Beckhaus et al., 2009) and can directly interact with β1 integrin *in trans* (Fiore, Ju et al., 2014). These facts suggest an important role of Thy-1 in drug resistance—indeed, high expression of breast cancer resistance protein (BCRP) has been identified in Thy-1 positive cells (Sukowati, Anfuso et al., 2013).

**FIGURE 1:**
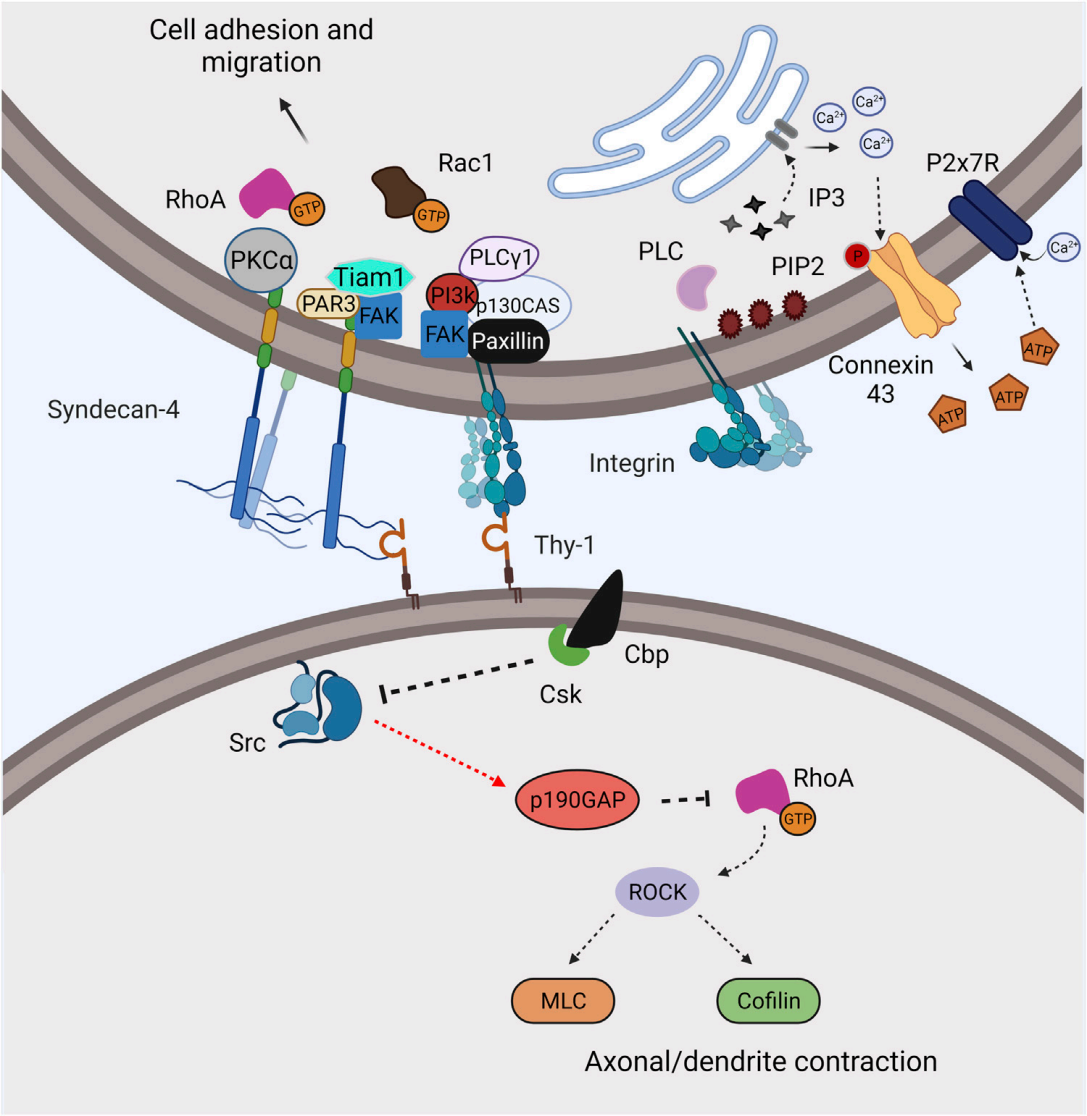
Thy-1 mediates and regulates integrin signaling through *trans*-interaction with integrins. Thy-1 can directly bind integrins and syndecan-4 and mediate distinctive signaling pathways between the two interacting cells. Recruitment of Cbp by Thy-1 to the lipid raft reduces Src activity and promotes contraction. On the other side, signaling induced by Thy-1–integrin/syndecan-4 interaction promotes adhesion and migration through canonical integrin outside-in signaling. The interaction also leads to elevated cellular Ca^2+^, opening of Connexin-43/Pannexin-1 hemichannels and activation of the P2X7R, resulting in astrocyte cell adhesion and migration.

Thy-1 induces a strong adhesion of reactive astrocytes to culture plates within the first hour of Thy-1 stimulation (Kong, Munoz et al., 2013), in a process dependent on Thy-1 binding to astrocytic αvβ3 integrin (Leyton, Schneider et al., 2001; Hermosilla, Munoz et al., 2008; Kong, Munoz et al., 2013). Additionally, in a different cellular model, the absence of Thy-1 (Thy-1^−/−^ cells) in fibroblasts induces faster migration than that observed for Thy-1^+/+^ cells, suggesting an inhibitory effect of Thy-1 on cell migration (Barker et al., 2004a). However, prolonged interaction between Thy-1 and αvβ3 integrin (>60 min) can promote astrocyte migration (Kong, Munoz et al., 2013), demonstrating that the initial block of cell migration mediated by Thy-1 disappears after the first hour of stimulation. The molecular mechanisms underlying the shift between strong cell adhesion and migration are still under study. Thy-1-astrocyte receptor binding results in the aggregation of αvβ3 integrin at the plasma membrane, the recruitment and phosphorylation of FAK and p130^Cas^, recruitment of vinculin, paxillin and PI3K (Leyton, Schneider et al., 2001; Kong, Munoz et al., 2013), as well as the activation of RhoA and p160ROCK (Avalos, Labra et al., 2002; Avalos, Arthur et al., 2004) and the inactivation of Rac1 (Kong, Munoz et al., 2013), events that lead to morphological changes and increased focal adhesion (FA) formation (Figure 1). The latter are points of adhesion of cells to the ECM, and enhanced number and area of these structures lead to stronger cell adhesion (Dubash, Menold et al., 2009). Moreover, syndecan-4, the latest identified member of the membrane proteoglycans is also involved in cell adhesion and migration (Akiyama, Yamada et al., 1989; Baciu and Goetinck 1995; Dovas, Yoneda et al., 2006). Importantly, syndecan-4 also acts as a receptor for Thy-1, and it is required in Thy-1-induced astrocyte adhesion and migration (Avalos, Valdivia et al., 2009; Kong, Munoz et al., 2013).

Pathways that are triggered in astrocytes by Thy-1-engagement of syndecan-4 have not been studied in detail; however, recent data indicates that upon Thy-1 binding, syndecan-4 regulates FA turnover in astrocytes and mouse embryonic fibroblasts by forming a complex with the PDZ-domain scaffold protein and regulator of cell polarity, PAR3 (Valdivia, Cardenas et al., 2020). This complex formation leads to dephosphorylation of FAK and activation of the Rac1GEF Tiam1 (Valdivia, Cardenas et al., 2020). Moreover, the cytoplasmatic domain of syndecan-4 has three tyrosine residues; one of them, Y180, is a phosphorylation site for the tyrosine kinase Src (Morgan, Hamidi et al., 2013). The mutation of Y180 for a lysine (Y180L) slows down FA dynamics, which in turn, reduces fibroblast migration by increasing cell adhesion (Morgan, Hamidi et al., 2013). This supports a role for syndecan-4 phosphorylation as a switch controller of FA assembly/disassembly dynamics and cell migration. Additionally, rapid FA turnover and migration induced by pY180-syndecan-4 is likely to occur due to the effect of syndecan-4 on integrin recycling (Brooks, Williamson et al., 2012; Morgan, Hamidi et al., 2013). Thus, the possibility that phosphorylation/dephosphorylation events of syndecan-4 could first promote adhesion and then lead to the attenuation of Thy-1–αvβ3 integrin-induced downstream pathways to decrease adhesion and increase cell migration is intriguing. On the other hand, evidence supports an additional role for syndecan-4 on PKCα activation in Thy-1-induced astrocyte adhesion, upstream of RhoA GTPase activation (Avalos, Valdivia et al., 2009). In addition, syndecan-4 mutated on Y188 (Y188L) disrupts this syndecan-4-mediated PKCα activation, and this residue is additionally involved in syndecan-4-mediated Rac1 activity (Bass, Morgan et al., 2007), as well as in integrin endocytosis (Bass, Morgan et al., 2007; Bass, Williamson et al., 2011). Therefore, Y188 also constitutes an important target to study integrin and syndecan-4 signaling pathway, particularly because of the involvement of PKCα and RhoA activation in astrocyte adhesion induced by Thy-1 (Figure 1).

The switch between astrocyte adhesion-migration appears to be controlled by differential activation of GTPases from the Rho family (Danen, van Rheenen et al., 2005). In addition, Thy-1–αvβ3 integrin engagement increases intracellular concentration of Ca^2+^, an event that occurs upstream of RhoA activation (Alvarez, Lagos-Cabre et al., 2016). Additional support for the involvement of the latter is that enhanced intracellular Ca^2+^ is blocked by IP3R-inhibitors, such as 2-APB and Xestospongin (Alvarez, Lagos-Cabre et al., 2016). This increase in Ca^2+^ is involved in hemichannel activation, which leads to ATP release, opening of P2X7R and a second and sustained increase in intracellular Ca^2+^ concentration, since inhibition of hemichannels (Connexins and Pannexins) abolishes these signaling events and astrocyte migration (Henriquez, Herrera-Molina et al., 2011; Alvarez, Lagos-Cabre et al., 2016, Figure 1). How does this IP3R-dependent Ca^2+^ increase activate hemichannels to induce ATP release? The answer to this question is currently under investigation.

### 2.3 The Thy-1, Syndecan-4 and Integrin Triplex in Signaling and Mechanosensing

The cellular responses mediated by Thy-1–integrin interaction are affected by mechanical forces. The interaction of Thy-1–integrin in the melanoma/EC model increases EC contraction, facilitating the extravasation of the melanoma cells through the endothelium (Schubert, Gutknecht et al., 2013). The mechanical tension generated by EC contraction could certainly affect melanoma cell adhesion, motility, and invasion (Bras, Radmacher et al., 2020); however, the effect of the mechanical forces exerted on the Thy-1–integrin interaction has not yet been studied on melanoma cells.

A similar molecular interaction between astrocytes and neurons triggers axonal contraction. Because these contractile forces are exerted on the Thy-1-ligated integrin of astrocytes (axonal pulling), astrocyte contraction is also detected, measured as FA and stress fiber (SF) formation, as well as by myosin light chain (MLC) phosphorylation (Perez, Rashid et al., 2021). FA and SF formation is enhanced because integrin levels are elevated when the cells are stimulated with Thy-1 plus mechanical stress (Perez, Rashid et al., 2021). Cell contraction induced as a consequence of the formation of these structures is thus exerted onto the substrate, where these cells attach, *i.e.*, the ECM. Therefore, axonal pulling promotes astrocyte contraction and the force generated is exerted on the matrix, where the cells adhere.

An additional component participates in regulating mechanosensing of the integrin in the Thy-1–integrin interaction. Syndecan-4 forms a trimolecular complex; the rigidity of the bonds in this complex changes in response to mechanical stress. At a single molecule level, the complex has a dynamic catch behavior when force is applied, and the molecular bonds change from a non-stiff to a stiff behavior required for cell adhesion. α5β1 integrin in A375 human melanoma cells and syndecan-4 form a trimolecular complex with Thy-1 associated surfaces (Beads) (Fiore, Ju et al., 2014). The bond behavior of the α5β1 integrin, syndecan-4 and Thy-1 complex in response to force does not change when signaling molecules such as dynamin or Src are inhibited, indicating that syndecan-4 signaling leading to integrin recycling is not involved in the bond behavior described. In this case, the mechanical forces that affect the extension of syndecan-4 glycosaminoglycan (GAG) chains change the conformation of the integrin, thereby leading to its activation (Fiore, Ju et al., 2014).

On the other hand, αvβ3 integrin and syndecan-4 in reactive astrocytes form a trimolecular complex with Thy-1-associated neurons. At a single molecule level, the complex displays a slip bond behavior when force is applied, and the molecular bonds are kept in a non-stiff phenotype required for cell contraction. This trimolecular interaction is mediated on the one hand, by the RLD motif of Thy-1 and the integrin, and on the other hand, by the heparin-binding domain of Thy-1 and syndecan-4. The interaction between syndecan-4 and Thy-1 is of lower affinity than that of α5β1 and αvβ3 integrins and Thy-1 (Burgos-Bravo, Martinez-Meza et al., 2020). Syndecan-4 promotes the functional effect of Thy-1–integrin on neurons, which is inhibited by Heparinase III or Heparin treatment, but this heparan sulfate blocking does not completely inhibit the effect. An additional role for the protein core is proposed by Leyton and co-workers (Burgos-Bravo, Martinez-Meza et al., 2020). However, could the integrin type, the cellular context, or the approaches used make the difference between these two experimental models? These are questions that still remain unanswered.

The cell machinery (Figure 1) involved in the biological effect of engaging Thy-1 in neurons through the binding of αvβ3 integrin includes the association of Thy-1 to a signal transducer molecule of the Transmembrane Associated Protein family, Csk binding protein (Cbp), the recruitment of the non-receptor tyrosine kinase Csk, the inactivation of Src, and the activation of the GTPase RhoA (Herrera-Molina, Frischknecht et al., 2012; Maldonado, Calderon et al., 2017). RhoA is a small G protein that regulates the actin cytoskeleton and cell contraction by targeting the effector protein ROCK, which elevates MLC phosphorylation and activation (Burridge and Wennerberg 2004). In the case of neurons, this signaling pathway includes MLC and cofilin phosphorylation, and leads to axonal retraction (Maldonado, Calderon et al., 2017). Syndecan-4 acts in conjunction with αvβ3 integrin to trigger Thy-1-induced neurite contraction (Burgos-Bravo, Martinez-Meza et al., 2020); however, its contribution to the signaling described above in neurite retraction, is yet to be investigated.

## 3 Thy-1 Mediates Integrin *cis*-regulation on the Plasma Membrane

### 3.1 Direct *cis*-Coupling of Thy-1 and Integrin αvβ3 Regulates Integrin Activity

The capability of Thy-1 to directly bind integrin in *cis* has long been explored (Barker et al., 2004b; Barker and Hagood 2009) but was only recently identified mechanistically (Fiore, Strane et al., 2015, Figure 2). Although Thy-1 showed a tendency to bind both αvβ3 and α5β1 integrins in their inactive conformation, its *cis*-interaction with αvβ3 has a profound impact on integrin downstream signaling, cell morphogenesis and pathogenesis (Fiore, Strane et al., 2015; Fiore, Wong et al., 2018). Like the aforementioned *trans*-interaction observed *in vitro* and *in vivo* (Leyton, Schneider et al., 2001; Herrera-Molina, Frischknecht et al., 2012; Lagos-Cabre, Alvarez et al., 2017; Burgos-Bravo, Figueroa et al., 2018), the direct *cis* coupling of integrin is dependent on the Thy-1 RLD motif. While the crystal structure of Thy-1 is not available yet, a predicted structure suggests that the RLD motif is located close to the protein’s N-terminal glycosylation site, facing outward from the anti-parallel structure of β-sheets that form an immunoglobulin (Ig)-like V type domain (Herrera-Molina, Valdivia et al., 2013) (UniProtKB—P04216). This effectively places the RLD motif no more than a few nanometers above the plasma membrane and thus, a *cis*-interaction with integrin can potentially further stabilize the heterodimeric protein in its bent, inactive conformation.

**FIGURE 2:**
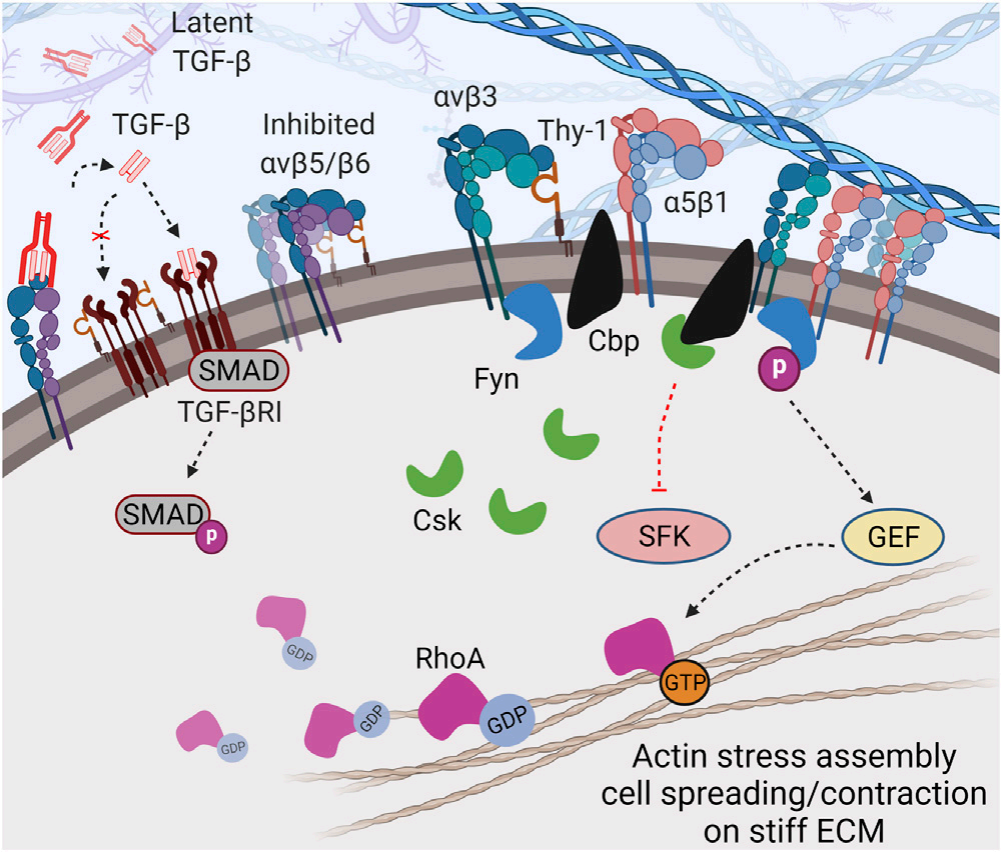
Thy-1 modulates cell mechanosensing through direct *cis*-interaction. Direct *cis*-coupling between Thy-1 and αv integrins (especially αvβ3) further stabilizes the integrin in bent-inactive conformation and simultaneously promotes recruitment of Cbp and Fyn to the cytoplasmic tail of the integrin. This leads to: 1) balanced signal transduction between α5β1 and αv integrins through competitive-cooperative binding to fibronectin, and 2) regulated activity of SFK due to Csk recruitment by Cbp to focal adhesions and Fyn-depended RhoA activation, which promotes cell spreading and contraction in the presence of mechanical stimuli (e.g., stiff matrices). In addition, Thy-1 can inhibit TGF-βRI and likely reduce αv integrin-dependent TGF-β activation (through αvβ5, αvβ6, and αvβ8), resulting in suppression of TGF-β signaling.

The Thy-1–αvβ3 integrin interaction physically couples the integrin to lipid raft microdomains containing critical signaling molecules to ensure proper mechanosensing. Fiore and others identified two proteins with critical regulatory roles in integrin signaling recruited to Integrin-Associated Complexes (IACs) through the Thy-1–integrin *cis*-interaction: Fyn, a member of the Src family kinases (SFK) and Cbp. Fyn has been identified as a critical player in sensing environmental rigidity and its activation is essential for translating extracellular mechanosignals into intracellular responses (Kostic and Sheetz 2006). This kinase is also responsible for integrin-mediated morphogenesis by activating downstream Rho GTPases (Liang, Draghi et al., 2004; Reddy, Smith et al., 2008). Cbp keeps SFK activity in check by recruiting Csk –the negative regulator of SFK– into IACs. Loss of Cbp has been shown to cause defective integrin mechanotransduction, resulting in impaired cell morphology (spreading) and migration (Shima, Nada et al., 2003). Interestingly, in nascent FAs, Fyn interacts with FAK and phosphorylates Cbp, resulting in subsequent recruitment/activation of Csk (Yasuda, Nagafuku et al., 2002; Maksumova, Le et al., 2005; Baillat, Siret et al., 2008), which in turn, negatively regulates local SFK activity and enables appropriate mechanotransduction.

Further downstream, Thy-1 has been shown to regulate cell spreading and SF assembly by promoting RhoA activity–likely due to downregulated c-Src-dependent p190 RhoGAP activity in the presence of Thy-1 (Barker et al., 2004a). Fyn has also been shown to directly phosphorylate and activate RhoGEF in response to integrin mediated force transduction, resulting in a more direct activation of RhoA (Guilluy, Swaminathan et al., 2011). Importantly, the activity of Rho GTPase is required for ECM stiffness-induced nucleus translocation of YAP/TAZ, which drives mechano-activation of fibroblasts and fibrosis (Dupont, Morsut et al., 2011; Liu, Lagares et al., 2015). Taken together, the direct and indirect regulatory role of Fyn on RhoA activity makes it a core modulator of force-induced cellular response.

### 3.2 Thy-1–Integrin *cis* Interaction Regulates Integrin Clustering

Thy-1 coupling with integrin can also suppress ligand binding-independent self-clustering of integrin and thus, reduce baseline integrin avidity, seen as reduced adhesion strength when cells are only allowed transient contact with the ECM (Fiore, Strane et al., 2015). Existing evidence has already shown that a tiny fraction of surface integrins can spontaneously switch into extended-active conformation without being activated –a thermodynamic nature of integrin (Li, Su et al., 2017). It is also known that integrin activation can be achieved simply through intramolecular interactions at the transmembrane domain between different integrin molecules (Ye, Kim et al., 2014). Therefore, the presence of Thy-1 can constraint self-clustering of integrin, indicating a higher-level regulatory role of Thy-1, other than suppressing activity at the single molecular level.

### 3.3 Thy-1 and Indirect Integrin Regulation in *cis*

In addition to direct *cis*-interacting with integrin and regulating recruitment of IAC components, Thy-1 can also indirectly regulate mechanotransduction through the TGF-β pathway. TGF-β-SMAD2/3/4 is well established as the main signaling route to induce mechano-related phenotypes and promote proliferation, contraction and ECM deposition (Vallee and Lecarpentier 2019). The role of TGF-β signaling in cancer and fibrosis has been well described (Budi, Schaub et al., 2021; Chung, Chan et al., 2021). As reported, Thy-1 null C57BL/6 mice were more prone to develop severe lung fibrosis after bleomycin treatment (Hagood, Prabhakaran et al., 2005), which could be a result of disrupted inhibitory coupling between Thy-1 and TGF-βRI (Koyama, Wang et al., 2017). Thy-1^−/−^ fibroblasts were more responsive to cytokines and growth factors like TGF-β, whereas Thy-1^+/+^ cells were resistant to similar treatments. The difference did not appear to be due to downstream signal transduction of TGF-β, but instead, to higher latent TGF-β activation in Thy-1^−/−^ cells (Zhou, Hagood et al., 2004). Likewise, induction of MMP9 by TGF-β has been observed in Thy-1^−/−^ fibroblasts, but not in Thy-1^+/+^ fibroblasts, implying that Thy-1 as an important suppressor in MMP9-induced latent TGF-β activation (Ramirez, Hagood et al., 2011). The interaction between Thy-1 and αvβ5 integrin has been proposed as a mechanism to constrain latent TGF-β activation by the integrin (Zhou, Hagood et al., 2010). The study, however, did not reveal if the inhibition was caused by *cis*- or *trans*-interaction between the two molecules. As Thy-1 can *cis* interact with β1 integrin, it is conceivable that Thy-1 is also capable of *cis*-interacting with other RGD integrins, including β5 and β6, and therefore, can maintain TGF-β activating αvβ5 and αvβ6 integrins in a low affinity conformation, reducing the activation of endogenous TGF-β (Figure 2). Indeed, in aging mice, TGF-β can effectively suppress Thy-1 expression at the transcriptional level by epigenetically inducing methylation of the Thy-1 promoter (Neveu, Mills et al., 2015), making the animals prone to fibrotic diseases.

*Trans* interaction between Thy-1 and syndecan-4 has been discussed; however, existing evidence also indicate a possible role of Thy-1 in integrin *cis-*regulation through syndecan-4 clustering. As an important co-receptor of fibroblast growth factor (FGF), syndecan-4 clustering and mobilization into lipid rafts can be induced by FGF treatment (Tkachenko and Simons 2002)—a phenomenon that could be similarly induced by the heparin-binding domain on Thy-1 through its interaction with the heparan sulfate chain on syndecan-4 (Elfenbein and Simons 2013). Noteworthy, syndecan-4 itself can sense environmental forces and mediate downstream signaling (Chronopoulos, Thorpe et al., 2020). Antonios used electromagnetic tweezers to apply repetitive, short (1 s) 1 nN tension pulses on syndecan-4, resulting in RhoA-dependent adaptive cell stiffening, whereas longer exposure (5 min) to a one-time small scale ∼200 pN force was sufficient to induce PI3K-mediated recruitment of Talin/Kindlin to FAs, with increased local β1 integrin activation and YAP nuclear translocation Chronopoulos, Thorpe et al. (2020). The evidence strongly suggests a *cis* correlation between syndecan-4 and β1 integrin during mechanotransduction. By coupling syndecan-4 to integrin, Thy-1 could indirectly contribute to integrin signaling when facing extracellular mechanical cues.

Another possible way for Thy-1 to regulate integrin activity could be by regulating the surface availability of the adhesion receptor. Reggie-1/Flotillin-2 has been shown to regulate integrin dynamics and FA turnover –loss of Reggie-1 promotes FA formation, FAK/Rac1 activity and integrin recycling (Hulsbusch, Solis et al., 2015). Interestingly, Reggie 1/2 colocalizes with Thy-1 and Fyn kinase on the plasma membrane, essentially marking that local lipid raft microdomain for internalization and therefore, reducing surface presence of proteins associated with Thy-1/Fyn (*i.e.*, integrins) (Lang, Lommel et al., 1998; Stuermer, Lang et al., 2001).

### 3.4 Thy-1 Fine Tunes Integrin Signaling Balance

Integrins are engaged by the ECM both cooperatively and competitively. This is particularly true for RGD integrins when they interact with the same RGD-motif on fibronectin. It is well established that during cell adhesion to fibronectin, a cooperative and synergistic crosstalk between α5β1 and αv integrins (especially αvβ3) is needed for mechanosensing, downstream signaling and cell spreading (Schiller, Hermann et al., 2013; Bharadwaj, Strohmeyer et al., 2017). α5β1 and αvβ3 clearly play different roles in mechanotransduction: αvβ3 is more of a “sensor” and could potentially reduce cellular contractility, making cells more pliable and invasive; whereas α5β1 functions as a force generator, responsible for adhesion strengthening and generation of contractility (Roca-Cusachs, Gauthier et al., 2009; Lin, Cohen et al., 2013; Milloud, Destaing et al., 2017; Strohmeyer, Bharadwaj et al., 2017).

In this context, it is critically important for cells to maintain a proper signaling balance between different integrins. As a *cis*-suppressor of αvβ3, Thy-1 could thus bear an essential role in maintaining this much needed signaling balance. *In vitro* studies (Fiore, Strane et al., 2015) have clearly demonstrated that Thy-1 can keep αvβ3 in bent-inactive conformation, reducing the reservoir of free integrins that can automatically switch into extended conformation under the thermodynamic equilibrium. Unsurprisingly, Thy-1 knockdown can significantly elevate αvβ3 signaling versus α5β1 signaling, and potentially drive fibroblasts into a myofibroblastic phenotype without environmental mechano stimulators (Fiore, Wong et al., 2018).

### 3.5 Other Potential Integrin *cis*-regulators With Functions Comparable to Thy-1

In addition to Thy-1, emerging evidence suggest other proteins can regulate integrin in *cis* as well. Semaphorin 7a (SEMA7a) is another GPI-AP capable of binding integrin *in trans* and deeply involved in TGF-β signaling and fibroblast differentiation (Kang, Lee et al., 2007; Suzuki, Okuno et al., 2007; Scott, McClelland et al., 2008; Esnault, Torr et al., 2017). SEMA7a could be another GPI-anchored membrane protein capable of interacting with integrin in *cis*, due to its function similarity to Thy-1.

While this review article focuses on Thy-1, a GPI-anchored protein in integrin *cis*-regulation, it is noteworthy that transmembrane proteins have also shown to regulate integrin activity in *cis* as well. One example is cell membrane metalloprotease ADAM17. The direct *cis-*interaction between ADAM17 and integrin α5β1 can keep both proteins in inactive form (Bax, Messent et al., 2004; Gooz, Dang et al., 2012; Grotzinger, Lorenzen et al., 2017) and this mutual inhibitory interaction can be enhanced by tetraspanin CD9 (Machado-Pineda, Cardenes et al., 2018), effectively adding another layer of regulation. Other examples include CD154 (also known as CD40L) and FcγRIIA, with their *cis* integrin binding promoting cell survival (CD154 and α5β1) (Bachsais, Salti et al., 2020) or inhibiting neutrophil recruitment (FcγRIIA and αMβ2) (Saggu, Okubo et al., 2018), respectively. It is plausible that in addition to well established inside-out and outside-in integrin regulations, a direct *cis*-interaction dependent mechanism also exists on the plasma membrane to regulate initial integrin-ECM binding. Comprehensive studies are needed to further reveal the extend and molecular details of such a mechanism.

## 4 Thy-1–Integrin Interaction in Pathophysiology

### 4.1 Thy-1 and Fibrosis

Fibrosis, defined as the excess accumulation of scar tissue composed of stiff, fibrillar ECM, leads to tissue and organ dysfunction. Fibrosis can lead to disfigurement (such as when it occurs in skin or joints), organ failure or death, and is often thought to be irreversible. Fibrosis can be driven by injury, inflammation, genetic variants, or aberrant mechanical stress on tissues, leading to aberrant and/or persistent activation of wound healing signaling paradigms. A myriad of cellular phenotypic alterations can initiate, accompany or amplify fibrosis, but the final common pathway always involves activated fibroblasts, which elaborate and/or remodel the fibrotic ECM. This is usually associated with phenotypic alteration of fibroblasts to a contractile myofibroblastic phenotype characterized by metabolic alterations, expression of contractile molecular machinery, excessive ECM production and resistance to apoptosis. Altered outside-in and inside-out integrin signaling is a key driver of fibroblast phenotype transitions; thus, molecules such as Thy-1, which modify integrin signaling in *cis* and in *trans* may offer opportunities for therapeutic interventions to slow, halt or even reverse fibrosis.

#### 4.1.1 Lung

Progressive pulmonary fibrosis (PF) has a high burden of morbidity and mortality. Idiopathic pulmonary fibrosis (IPF), the most prevalent form, has no cure and inevitably results in death or lung transplantation. FDA-approved antifibrotic drugs are expensive, fraught with uncomfortable side effects, and only slow disease progression, with minimal effects on mortality (Moua and Ryu 2019). A critical barrier in the field is a lack of understanding of the regulatory paradigms controlling the emergence, function, and resolution of fibroblast phenotypes in PF.

Differentiation and activation of myofibroblasts and lipid-containing lipofibroblasts is critical for the development of pulmonary alveoli (where oxygen enters the bloodstream) and for wound repair and fibrosis (Kis, Liu et al., 2011; Li, Li et al., 2015; Branchfield, Li et al., 2016; El Agha, Moiseenko et al., 2017). Cell surface Thy-1 expression is important in both processes. Thy-1 null mice have abnormal alveolar development (Nicola, Hagood et al., 2009) and more severe lung fibrosis (Hagood, Prabhakaran et al., 2005), which fails to resolve (Liu, Wong et al., 2017). In human IPF, Thy-1, which is normally expressed by most human lung fibroblasts, is absent in fibroblastic foci (FF) (Hagood, Prabhakaran et al., 2005), which are abnormal collections of myofibroblasts driving fibrosis progression. Thy-1 is silenced within FF by epigenetic mechanisms, such as DNA methylation (Sanders, Pardo et al., 2008). Epigenetic silencing of Thy-1 leading to pathogenic alteration in fibroblasts, is driven by aging and TGF-β (Neveu, Mills et al., 2015), TLR4 (Xing, Nie et al., 2015), IL-17 (Neveu, Staitieh et al., 2019) and hypoxia (Robinson, Neary et al., 2012). A recent study described a regulatory axis of Thy-1 expression involving the transcription factor YY1 and miR-214 in the context of lung fibrosis (Chen, Yang et al., 2020).

From a functional perspective, Thy-1 has been shown to regulate many core functions and phenotypic features of fibroblasts relevant to fibrogenesis, including proliferation, cytokine and growth factor expression and responsiveness, adhesion, migration, myofibroblast/lipofibroblast differentiation, and cell survival. Thy-1 expression is lost in the transition from fibroblasts to myofibroblasts (Hagood, Guo et al., 1999; Hagood, Mangalwadi et al., 2002; Barker et al., 2004a; Zhou, Hagood et al., 2004; Hagood, Prabhakaran et al., 2005; Rege, Pallero et al., 2006; Sanders, Kumbla et al., 2007; Varisco et al., 2012; Liu, Wong et al., 2017). The expression of Thy-1 supports a lipofibroblast phenotype, which is important in normal lung alveolar development (McQualter, Brouard et al., 2009; Varisco et al., 2012; McQualter, McCarty et al., 2013) and its absence supports a contractile myofibroblast phenotype, which activates latent TGF-β1 and resists apoptosis (Zhou, Hagood et al., 2004; Sanders, Kumbla et al., 2007; Liu, Wong et al., 2017). Prenatal tobacco smoke exposure promotes Thy-1 DNA methylation/silencing in embryos, predisposing mice to lung fibrosis in adulthood (Cole, Brown et al., 2017).

Integrin αv signaling is a key regulator of fibrosis in multiple organs (Henderson, Arnold et al., 2013; Sun, Chang et al., 2016). IPF fibroblasts lacking Thy-1 expression, demonstrate persistent activation of mechanosensitive integrin signaling, regardless of whether they are in a mechanically soft environment (like normal lung alveoli) or a mechanically stiff environment (like established fibrotic tissue). Conversely Thy-1 expression inhibits activation of SFK, RhoA, and downstream myofibroblast differentiation pathways in soft environments (Fiore, Strane et al., 2015), including FF, which have mechanical properties similar to normal alveolar regions (Fiore, Wong et al., 2018). Thus, integrin activation in the Thy-1-negative myofibroblasts that populate FF likely initiate a cycle of matrix remodeling that leads to progression and persistence of fibrosis (Fiore, Wong et al., 2018). A single lung injury, such as that induced experimentally by administration of intratracheal bleomycin in mice, leads to transient, reversible loss of cell surface Thy-1 and fibrosis that spontaneously resolves. Repetitive injury, however, leads to silencing of Thy-1 at the mRNA level and persistent activation of αv integrin, resulting in progressive, non-resolving fibrosis (Tan, Jiang et al., 2019).

Evidence suggests that Thy-1 modulation of profibrotic signaling can be harnessed for therapeutic benefit. In a human cytomegalovirus-induced model of acute interstitial pneumonia (AIP), characterized by a rapid-onset form of fibrosis associated with acute lung injury, lentiviral transfection of Thy-1 partially attenuated fibrosis, by blocking WNT activation (Chen, Tang et al., 2019). Exogenous soluble Thy-1-Fc fusion protein (sThy-1) blocked TGF-β1 activation and reversed the myofibroblast phenotype *in vitro* (Zhou, Hagood et al., 2004). Recent studies in two distinct models of PF, induced either by bleomycin injury or by transgenic expression of active TGF-β1, demonstrated that intravenous administration of sThy-1, but not the non-integrin-binding RLE form [sThy-1 (RLE)], is able to reverse established fibrosis *in vivo* (Tan, Jiang et al., 2019). Extracellular vesicle-based therapeutics are widely studied in models of fibrosis in multiple organs and tissues, and in a variety of clinical trials. The ability of mesenchymal stem cell (MSC)-derived extracellular vesicles to reverse myofibroblast differentiation in lung fibroblasts is attributable to Thy-1 modulation of integrin activation (Shentu, Huang et al., 2017). Fibrosis-suppressive functions of Thy-1 have been demonstrated in other organs as well.

#### 4.1.2 Liver

Fibrosis of the liver, also known as cirrhosis, causes one million deaths worldwide (Asrani, Devarbhavi et al., 2019), and liver is the second most common solid organ transplant, usually because of cirrhosis. In a mouse model of liver fibrosis induced by bile duct ligation, absence of Thy-1 increases fibrosis severity (Nishio, Koyama et al., 2021). In liver portal fibroblasts, Thy-1 forms an inhibitory complex with TGF-βR1 that is disrupted by mesothelin (Msln), in a mechanism also involving Muc16 (Koyama, Wang et al., 2017).

#### 4.1.3 Kidney

Chronic kidney disease involving fibrotic remodeling is widespread and requires management with chronic dialysis or transplantation. A widely used model of kidney inflammation leading to fibrosis is injection of antibodies to Thy-1 in mice, leading to mesangial cell injury (Becker and Hewitson 2013). In a unilateral ureteral obstruction model of kidney injury, Thy-1-null mice exhibit increased fibrosis severity, *via* similar mechanisms to the bile duct ligation model in liver (Nishio, Koyama et al., 2021).

#### 4.1.4 Heart

Congestive heart failure, which usually involves myocardial fibrosis, is a major cause of mortality worldwide. In a myocardial infarction model of cardiac fibrosis, transplantation of Thy-1^+/+^ cardiac fibroblasts accelerated resolution of fibrosis and improved repair (Chang, Li et al., 2018). On the contrary, absence of Thy-1 increases severity of cardiac fibrosis in a transverse aortic constriction model (Li, Song et al., 2020).

#### 4.1.5 Joints

The importance of Thy-1 in fibroblast phenotype has recently been confirmed in arthritis (Croft, Campos et al., 2019), in which Thy-1^+/+^ fibroblasts regulate inflammation, whereas Thy-1^−/−^ fibroblasts mediate bone and cartilage remodeling.

#### 4.1.6 Summary

In multiple tissues and organs, in a wide variety of models of fibrosis and in several chronic human diseases, Thy-1 modulates development and progression of fibrosis. Its absence, usually due to epigenetic silencing associated with aging and/or inflammation, worsens fibrosis. Mechanistically, the fibrosis-suppressive functions of Thy-1 involve modulation of integrin activation and signaling, although notably, Thy-1 can also affect TGF-β and WNT signaling.

### 4.2 Thy-1 and Other Normal Cell Differentiation and Determination

#### 4.2.1 Thy-1 and Stem Cell Differentiation and Function

Thy-1 has long been known as a marker of hematopoietic (Boswell, Wade et al., 1984) and MSCs (Ghilzon, McCulloch et al., 1999). It is unclear, however, the degree to which Thy-1, either *via* integrin modulation or other molecular interactions, mediates or modulates stem cell pluripotency or multipotency. Interestingly, in reprogramming fibroblasts to create induced pluripotent stem cells (iPSCs), Thy-1 is one of the first somatic markers to be repressed at the initiation of reprogramming (Li, Dang et al., 2014). Reprogrammed cells that either retain or regain Thy-1 expression lack true pluripotency and retain/regain a mesenchymal phenotype, in part through miRNA regulation of Wisp1 and genes regulating cell-ECM interactions and growth factor signaling.

Differential expression of Thy-1 in subsets of adipose-tissue derived MSCs (or stromal cells) affects their proliferation and metabolism, in part through activation of AKT (Pan, Zhou et al., 2019). Thy-1 promotes osteogenic (vs. adipogenic) differentiation of MSCs (recently reviewed in Saalbach and Anderegg 2019). This pro-osteogenic role of Thy-1 is dependent on β3, but not β1 integrin, and interestingly can be mediated by Thy-1 in both *cis* and *trans*, and was associated with stimulation of the canonical *Wnt* signaling pathway (Picke, Campbell et al., 2018). The same study demonstrated higher body fat mass and lower bone density in Thy-1^−/−^ mice. A different study which used shRNAs to silence Thy-1 expression in MSCs showed increased adipogenic and osteogenic differentiation, indicating that Thy-1 functions as a differentiation obstacle (Moraes, Sibov et al., 2016). Thy-1 expression is itself decreased during adipogenesis *via* epigenetic silencing (Flores, Woeller et al., 2019). Thy-1 inhibits adipogenesis in preadipocytes *via* inhibition of Fyn and PPARγ (Woeller, O'Loughlin et al., 2015). PPARγ activity can also suppress Thy-1 expression *via* microRNA (miR) 103 (Woeller, Flores et al., 2017). Conversely, in lung fibroblasts, Thy-1 promotes PPARγ signaling and lipid accumulation (Varisco et al., 2012). Others have also shown that Thy-1 expression supports lipofobroblast differentiation of lung fibroblasts (McQualter, McCarty et al., 2013). This is also true of Sca1^+/+^ mesenchymal progenitors in the transition from saccular to alveolar stages of lung development; Thy-1^high^ cells preferentially generate Oil Red O-positive lipofibroblasts, whereas their Thy-1^−/low^ counterparts generate an interconnected network of non-lipofibroblastic cells (McQualter, Brouard et al., 2009). Thus, the effects of Thy-1 on regulating lipid metabolism signaling and stemness/pluripotency are very complex and likely depend on the cellular context.

An increasingly appreciated function of MSCs is their paracrine effects mediated through extracellular vesicles, which can be harnessed for cell-based therapy. Human MSC-derived extracellular vesicles have emerged as a new therapeutic strategy for many diseases (van der Pol, Boing et al., 2012; Fais, O'Driscoll et al., 2016). Extracellular vesicles are comprised of mRNAs, non-coding RNAs, proteins and membrane lipids derived from donor cells. Extracellular vesicles can modulate cell proliferation, tissue repair, and regeneration (Biancone, Bruno et al., 2012; Lamichhane, Raiker et al., 2015). Several routes of extracellular vesicle uptake have been shown in different cell types (Mulcahy, Pink et al., 2014) and this uptake is repressed by endocytosis pathways (Svensson, Christianson et al., 2013; Tian, Li et al., 2014). Thy-1 has been shown to mediate uptake of viral particles by glioblastoma cells and fibroblasts (Li, Fischer et al., 2016). Interaction of Thy-1 with β integrins mediates MSC-derived extracellular vesicle uptake by lung fibroblasts, which blocks myofibroblastic differentiation; MSC-derived extracellular vesicles are enriched for miRs that target profibrotic genes upregulated in IPF fibroblasts (Shentu, Huang et al., 2017). Blocking the RGD-binding β integrin “partners” of αv (β1, 3 and 5), as well as silencing them in recipient cells, blocks the uptake of MSC-derived extracellular vesicles by myofibroblasts.

#### 4.2.2 Thy-1 and Immunity

Thy-1 was originally described as a lymphocyte marker (Reif and Allen 1964b). Despite extensive studies over decades on the role of Thy-1 in augmenting T cell signaling, its exact role in immunity has remained somewhat enigmatic (Haeryfar and Hoskin 2004; Furlong, Power Coombs et al., 2017). Most of the Thy-1-associated signaling in immune cells requires crosslinking of Thy-1 or engagement of other receptors, such as the T-cell receptor (TCR), but does not involve integrin interactions. A number of Thy-1^+/+^ mesenchymal or stromal cells modulate T cell function, but it is still unclear whether Thy-1 plays a direct role in such interactions (Kitayama, Emoto et al., 2014; Pfisterer, Lipnik et al., 2015).

#### 4.2.3 Thy-1 and Vascular Biology

Thy-1 is expressed on activated ECs and interacts with leukocyte integrins *in trans* to regulate inflammatory cell vascular adhesion and transmigration (Wetzel, Chavakis et al., 2004). The recruitment and extravasation of multiple different types of leukocytes, including neutrophils, monocytes and eosinophils, into inflamed sites, such as lung and peritoneum, is impaired in Thy-1-null mice, and is not rescued by transplantation of wild type bone marrow, indicating that endothelial Thy-1 expression is required for optimal leukocyte recruitment (Schubert, Polte et al., 2011). Likewise, endothelial Thy-1 expression is required for melanoma cell metastasis (Schubert, Gutknecht et al., 2013). Thy-1 is expressed on lymphatic vessels in the lung (Kretschmer, Dethlefsen et al., 2013), but the functional significance of this expression is unknown.

### 4.3 Thy-1 and Cancer

Thy-1 has complex and multiple roles in different types of cancer, as detailed in excellent reviews (Kumar, Bhanja et al., 2016; Sauzay, Voutetakis et al., 2019). Briefly, Thy-1 functions as a tumor suppressor in multiple malignancies, including nasopharyngeal and ovarian cancer (Lung, Bangarusamy et al., 2005). This function appears dependent on the interaction with β3 integrin (Chen, Hsu et al., 2016). Loss of heterozygosity (LOH) at 11q23.3-q24.3, where *THY1* is mapped in humans, is associated with poor prognosis for ovarian cancer (Cao, Abeysinghe et al., 2001). As reported, forced Thy-1 expression suppresses tumorigenicity in the ovarian cancer cell line SKOV-3 (Cao, Abeysinghe et al., 2001; Abeysinghe, Cao et al., 2003). In neuroblastoma, Thy-1 expression correlates inversely with prognosis (Fiegel, Kaifi et al., 2008). On the other hand, Thy-1 functions as a cancer stem cell marker in several types of malignancy (Shaikh, Kala et al., 2016). In liver cancer, for example, Thy-1 promotes tumor progression in part *via* β3 integrin interaction (Chen, Chang et al., 2015).

## 5 Discussion and Future Perspective

Many members of IgSF are identified as cell adhesion molecules (CAMs) and many of them have shown important role in cancer metastasis and neuron development (Crossin and Krushel 2000; Wai Wong et al., 2012). Among them, Thy-1 with its unique role in integrin-mediated multiple cell-matrix and cell-cell interactions truly stands out. Upon ligand binding, these receptors trigger a myriad of divergent intracellular signaling events *in cis* and *in trans* that control the actin cytoskeleton and affect cellular processes, such as adhesion, migration, and ECM remodeling (Table 1).

**TABLE 1:** The interaction of Thy-1 with integrins in *trans*- or in *cis*-results in diverged impacts on downstream integrin signaling.

Interaction type	*Trans*	*Cis*
FAK	Activating	Increasing sensitivity
c-Src	Inhibiting	Inhibiting
Fyn	Unidentified	Recruiting
RhoA	Activating	Increasing sensitivity
Rac-1	Inhibiting	Unidentified
MMP9	Inducing production	Reducing production by inhibiting TGF-β

Integrins bind to Thy-1, thus enlarging Thy-1 clusters. As a GPI-anchored protein, Thy-1 spontaneously forms small nanoclusters in the plasma membrane, and its ligand binding reportedly induces protein aggregation. Such reduction of mobility and clustering of Thy-1 could be facilitated by interaction between Thy-1 molecules, Thy-1–integrin binding, the association between Thy-1 and Cbp in lipid rafts, and the interaction of the Thy-1 membrane complex with the cortical cytoskeleton. By means of this protein aggregation process, Thy-1 also promotes integrin clustering in their inactive/active form, thus regulating integrin function.

Although lacking a cytoplasmic tail, Thy-1 mediates a large variety of integrin-related signaling pathways in a context-dependent manner. When binding with integrin *in trans*, Thy-1 functions as a generic ligand for the molecule, promoting cell-cell association and integrin outside-in signaling. On the mechanical side, the trio of Thy-1, integrin and syndecan-4 works synergistically and generates rapid binding strengthening with catch bond characteristics. However, binding with integrin *in cis* plays vastly distinctive roles from the *trans*-interaction, albeit with the same partner. Instead of mediating integrin outside-in signaling, the *cis*-interaction between Thy-1 and integrins further stabilizes the adhesion receptor in the inactive, bent conformation, thus suppressing auto-activation of integrins, which is a nature of its thermodynamic sway between different conformational states. In addition to direct inhibition of integrin activity, Thy-1 also brings together lipid raft-tethered signaling proteins, especially Fyn and Cbp, to the proximity of integrins. This pre-assembled protein complex is critical for integrin-mediated mechanotransduction —Fyn enables fast cellular mechanical responses and RhoA-dependent force generation, whereas Cbp recruits the SFK inhibitor Csk to the site and ensures that the signaling is tightly regulated. Through suppressing TGF-β-activating integrins (αvβ5 and αvβ6) and TGF-βR1, Thy-1 indirectly downregulates TGF-β signaling, which could have an even more significant biological impact on its integrin regulatory role.

**FIGURE 3:**
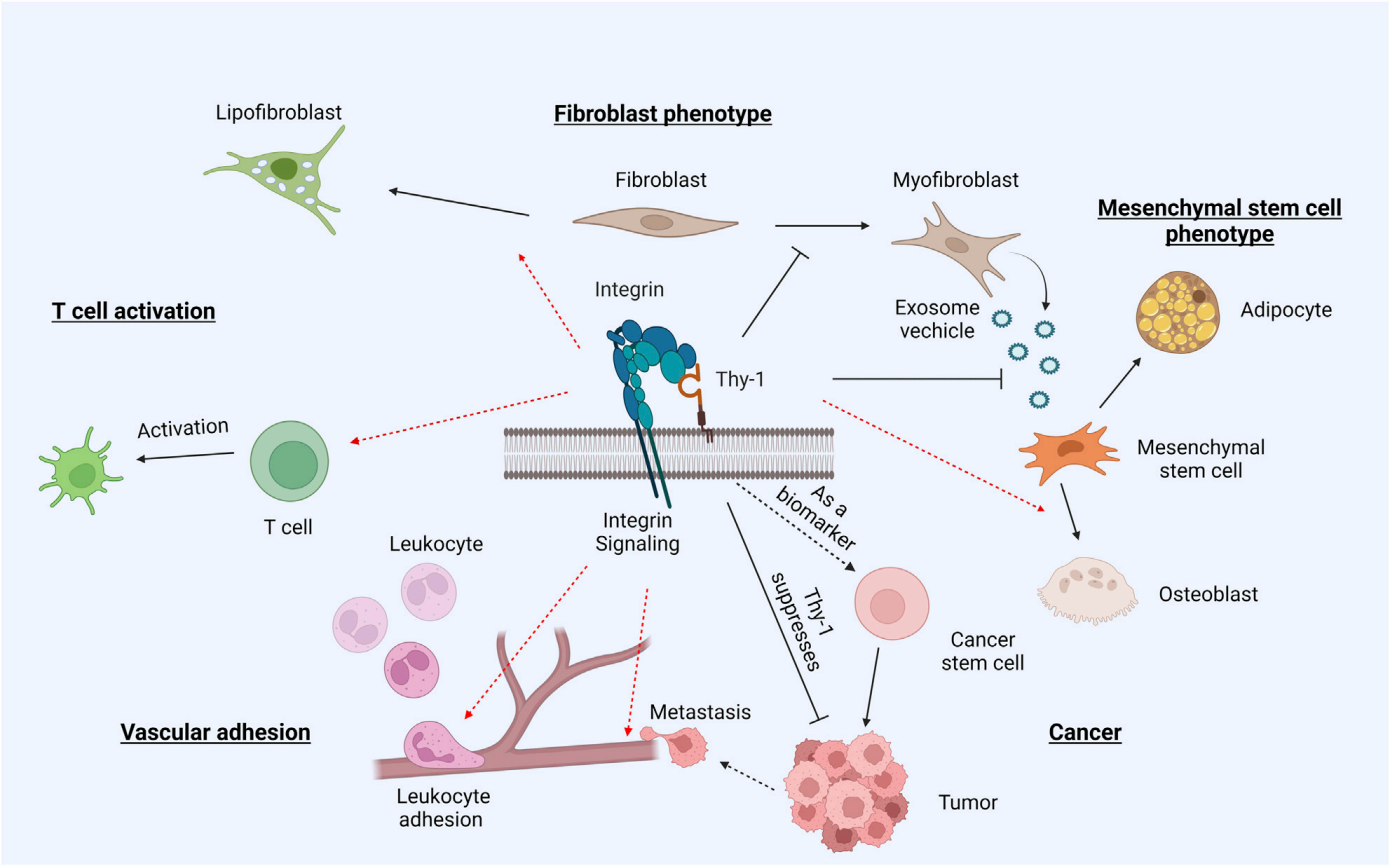
Thy-1 and integrin in pathophysiology. Through binding and regulating integrins, Thy-1 plays an intricate role in differentiation and tumorigenesis. The presence of Thy-1 promotes differentiation of naïve fibroblasts into lipofibroblasts, but suppresses myofibroblast activation. Similarly, Thy-1 drives MSC differentiation into osteoblasts, while inhibiting differentiation into adipocytes. The Thy-1–integrin interaction also promotes T cell activation and leukocyte adhesion. In cancer, Thy-1 displays somewhat contradictory roles –often regarded as an oncogene, but also promoting metastasis through its *trans* interaction with integrins.

Apart from RGD integrins (αv and α5β1 integrins), other integrins, namely αMβ2 and αXβ2, that likely cannot directly bind the Thy-1 RLD motif, have also shown interactivity with Thy-1. This is particularly interesting, since it suggests that Thy-1 could be a pan-integrin regulator with additional unidentified biological impacts. This implication is actually well aligned with the perplexing pathophysiological role of Thy-1 *in vivo* (Figure 3). While it universally acts as a suppressor in fibrotic diseases across various organs, Thy-1 bears a much more complicated role in cancer biology —it is considered as a tumor suppressor in some cases, but also correlates with poor prognosis and increased metastasis in some other types of cancer.

For an extensive period following its initial discovery, Thy-1 had been widely described as a surface marker for cell differentiation and tissue development, particularly in T cells. However, increasing evidence has collectively demonstrated that Thy-1, through its capability of interacting with integrins *in trans* and *in cis*, plays a much broader biological role. To make the underlying mechanistic network even more intricate, as a lipid raft GPI-anchored protein, Thy-1 can dynamically bring together multiple membrane proteins to regulate/mediate downstream spatial-temporal signaling and hence, guide cellular responses in an ever-changing microenvironment. In order to fully unveil the true nature of Thy-1 (and to a broader extent, lipid raft-anchored integrin regulators), a combination of next generation sequencing techniques (high-throughput RNA sequencing, single cell RNA sequencing and ATAcSeq, etc.) with proteomics studies and advanced microscopy is needed. Only then will we be able to have a more comprehensive glimpse of how integrins, together with other transmembrane proteins, actively sense and transmit signaling across the plasma membrane, decoding relevant environmental cues into distinctive intracellular signaling events —with Thy-1 occupying a critical place in the process.

## Glossary

Thy-1: Thymus cell antigen one
CD90: Cluster of differentiation 90
BRCP: Breast cancer resistance protein
EC: Endothelial cells
ECM: Extracellular matrix
ECM1: Extracellular matrix protein1
HDMEC: Human dermal microvascular endothelial cells
GAG: Glycosaminoglycan
GPI: glycosylphosphatidylinositol
FA: Focal adhesion
FAK: Focal adhesion kinase
FGF: Fibroblast growth factor
TGF-β: Transforming growth factor β
PLC: Phospholipase C
DC: Dendritic cells
MMPs: Matrix metalloproteinases
TNF: Tumor necrosis factor
TCR: T-cell antigen receptor
LAP: Latency-associated peptide
CAFs: Cancer-associated fibroblasts
CAMs: Cell adhesion molecules
MLC: Myosin light chain
Cbp: Csk binding protein
IACs: Integrin-Associated Complexes
IgSF: Immunoglobin superfamily
SFK: Src family kinases
SEMA7a: Semaphorin 7a
PF: Pulmonary fibrosis
IPF: Idiopathic pulmonary fibrosis
FF: Fibroblastic foci
MSC: Mesenchymal stem cell
Msln: Mesothelin
YAP: Yes-associated protein
